# A clinically applicable CKD diagnostic model derived from sound touch viscosity ultrasound and LASSO regression

**DOI:** 10.3389/fbioe.2025.1651500

**Published:** 2025-09-22

**Authors:** Wei Zhu, Xingyu Wang, Bin Xia, Xin Wang, Jianke Chen, Xinyu Fu, Jian Chen

**Affiliations:** Department of Ultrasound in Medicine, The Fourth Affiliated Hospital of School of Medicine, and International School of Medicine, International Institutes of Medicine, Zhejiang University, Yiwu, China

**Keywords:** chronic kidney disease, sound touch viscosity, diagnostic model, nomogram, machine learning

## Abstract

**Background:**

chronic kidney disease (CKD) remains a global health challenge with limitations in current diagnostic methods, including the invasiveness of biopsies and variability of estimated glomerular filtration rate (eGFR). This study aimed to develop a noninvasive diagnostic model integrating ultrasound viscoelasticity parameters to address these gaps.

**Methods:**

A prospective cohort of 228 participants underwent standardized renal ultrasound with viscoelastic imaging (Mindray Resona A20) to assess viscoelastic parameters and structural metrics. Key predictors were selected through LASSO regression, and a logistic regression diagnostic model was constructed. Model performance was comprehensively evaluated by analyzing discriminative ability (AUC, sensitivity/specificity), calibration (Brier score, calibration curves), and clinical utility (nomogram development, risk stratification and stage-specific decision curve analysis). Multiclass analysis was implemented to evaluate stage-specific performance (Class 1: normal; Class 2: G1-3; Class 3: G4-5) using one-vs-rest ROC methodology. All statistical analyses incorporated 1000 bootstrap iterations for robust variance estimation.

**Results:**

The diagnostic model demonstrated superior accuracy with an AUC of 0.932 (95% CI 0.908-0.956) in validation sets. Pathological analysis revealed that viscosity values were significantly elevated in CKD patients compared to controls (1.99 vs. 1.64 Pa·s, *P* < 0.001), while elasticity and shear wave velocity showed increases of 12.7%-13.2% and 5.3% respectively (*P* < 0.001). For clinical implementation, the model incorporated a visual nomogram that converted scores ranging from 0 to 160 points into CKD probability estimates between 0.1 and 0.9, with an optimal cutoff value of 0.383 providing balanced sensitivity of 88.4% and specificity of 87.8%. Decision curve analysis confirmed clinical utility across probability thresholds of 20%-80%, with peak net benefit at 40% threshold probability. Multiclass analysis revealed stage-dependent performance: Class 3 showed the highest discrimination (AUC = 0.918), followed by Class 1 (AUC = 0.884) and Class 2 (AUC = 0.774), with significant inter-stage differences (DeLong’s test *P* < 0.001).

**Conclusion:**

This study establishes a novel “function-structure” integrated diagnostic paradigm for CKD, combining the accuracy of ultrasound parameters with unique structural insights. The model’s noninvasive nature and stability under physiological variability make it particularly valuable for early detection and longitudinal monitoring.

## 1 Introduction

Chronic kidney disease (CKD) currently ranks among the top ten leading causes of death worldwide (Webster et al., 2017). Epidemiological projections indicate that by 2040, this disease will rise to become the fifth leading global cause of mortality (Foreman et al., 2018). Thus, the development of novel monitoring and diagnostic approaches has become increasingly critical (Ruiz-Ortega et al., 2020; Thomas et al., 2017).

In clinical practice, early diagnosis and intervention are crucial for improving patient outcomes. However, current assessment methods present significant limitations: renal biopsy is invasive, unsuitable for repeated monitoring, and may lead to various complications including hemorrhage (Whittier and Korbet, 2004); while the commonly used estimated glomerular filtration rate (eGFR) does not consistently correlate with fibrosis severity and lacks sensitivity for subclinical injury (Ruiz-Ortega et al., 2020). The 2024 KDIGO (Madero et al., 2025) guidelines reinforce albumin-to-creatinine ratio (ACR) as a cornerstone for CKD risk stratification, yet ACR exhibits limited sensitivity in early-stage CKD. Consequently, noninvasive approaches are essential for active CKD monitoring as alternatives to repeated biopsies and to complement the diagnostic gaps of eGFR and ACR in early disease detection. Renal ultrasonography has been widely adopted in clinical evaluation due to its safety profile, primarily through measurements of kidney size and cortical thickness to assess renal function (Tuma, 2013; Yamashita et al., 2015). Nevertheless, this modality suffers from two major limitations: operator-dependent variability in results, and the frequent manifestation of abnormal findings only in advanced disease stages. Studies have demonstrated the restricted value of conventional ultrasonography in early CKD diagnosis (Sicari et al., 2013; Diabetic et al., 2005; Initiative, 2007).

Meanwhile, noninvasive quantitative assessment of tissue mechanical properties represents a critical advancement in medical imaging. As an innovative ultrasound technique, shear wave elastography (SWE) enables *in vivo* quantification of tissue stiffness and has emerged as a research focus in medical imaging over the past decade. This modality has been recommended by the World Federation for Ultrasound in Medicine and Biology (WFUMB) and European Federation of Societies for Ultrasound in Medicine and Biology (EFSUMB) guidelines, while receiving approval from the U.S. Food and Drug Administration (FDA). Currently, SWE has been extensively applied in disease diagnosis across multiple organs including liver and breast tissues (Ferraioli et al., 2015; Barr et al., 2015; Ferra et al., 2018).

Notably, the mechanical properties of biological tissues encompass not only elasticity but also viscosity. Tissue viscosity not only influences the measurement accuracy of elastic parameters but also serves as an independent biomechanical marker with significant clinical value. Emerging evidence demonstrates a strong correlation between tissue viscoelastic parameters and inflammatory activity, a finding that has shown promising applications across multiple domains including liver fibrosis assessment and differential diagnosis of breast tumors (Ferraioli et al., 2015; Barr et al., 2015; Ferra et al., 2018). As an essential complement to conventional elastography, viscoelasticity measurement technology is emerging as a novel research direction in tissue biomechanics and has garnered substantial attention from the international academic community.

The sound touch viscosity (STVi) can be calculated based on shear wave elastography by extracting shear waves at different frequencies to obtain frequency-dependent shear wave velocities, followed by applying a viscoelastic fitting model to derive viscosity coefficients and dispersion coefficients, thereby enabling noninvasive assessment of tissue viscoelasticity. In recent years, quantitative tissue viscoelasticity techniques have demonstrated significant value in evaluating diseases across multiple organ systems. Clinical applications in liver fibrosis staging (Yin et al., 2007; Ziol et al., 2005) and differential diagnosis of breast/thyroid nodules (Jia et al., 2024; Lee et al., 2019; Kim et al., 2024; Stoian et al., 2023) have confirmed that ultrasound viscoelastic imaging can effectively distinguish mechanical property differences between pathological and normal tissues. Particularly, the STVi technique based on shear wave dispersion analysis quantifies the frequency dependence of shear wave propagation velocity to simultaneously obtain tissue elastic modulus and dynamic viscosity parameters, with its reliability validated in multicenter studies (Jia et al., 2024; Mao et al., 2025). However, two critical gaps remain in applying this technology to chronic kidney disease (CKD): First, systematic studies are lacking on the correlation between viscoelastic parameters and the degree of renal dysfunction; Second, no diagnostic criteria for CKD staging have been established based on viscoelastic characteristics.

Building upon this foundation, the present study proposes an innovative multimodal assessment strategy: by integrating conventional renal ultrasound parameters with ultrasound viscoelastic imaging (Voigt model-derived Vi Mean and E Mean) (Chen et al., 2009; Kanai, 2005) and demographic characteristics (age, sex, BMI), we have developed an interpretable machine learning diagnostic model. This model not only preserves the clinical utility of traditional indicators but also leverages viscoelastic parameters to reflect microstructural changes in renal tissue, potentially addressing the clinical challenge of insufficient sensitivity in early CKD diagnosis. The research outcomes will provide a novel tool for noninvasive and dynamic monitoring of renal function while advancing the application of precision medicine in nephrology.

## 2 Methods

### 2.1 Patients

This study employed a prospective single-center cohort design to develop and validate a predictive diagnostic model for chronic kidney disease (CKD) based on ultrasound viscoelastic imaging. The study cohort comprised consecutive CKD patients admitted to the Department of Nephrology at the Fourth Affiliated Hospital of Zhejiang University School of Medicine between September 2024 and March 2025. The research protocol was approved by the Fourth Affiliated Hospital of Zhejiang University School of Medicine (K2025134), and written informed consent was obtained from all participants. The study strictly adhered to the KDIGO Clinical Practice Guidelines for CKD diagnosis (Stevens and Levin, 2013). The sample size estimation was based on a 9.1% prevalence rate of the disease in the population, with an anticipated 20% exclusion rate of cases. This study used “chronic kidney disease (CKD) status” as the primary endpoint (dichotomous outcome: 1 = CKD, 0 = non-CKD). To detect an association strength of an odds ratio = 2.0 between the target exposure factor and CKD (α = 0.05, power = 80%), the sample size was calculated based on a case-control study design using a logistic regression model framework for dichotomous outcomes. The calculation was performed using PASS 15.0 statistical software, which indicated a requirement of 140 CKD patients to be included. The inclusion criteria required participants to demonstrate either persistent renal dysfunction, defined as two consecutive measurements taken at least 12 weeks apart showing an estimated glomerular filtration rate <90 mL/min/1.73 m^2^, or significant proteinuria indicated by an albumin-to-creatinine ratio (ACR) of at least 30 mg/g or a protein-to-creatinine ratio (PCR) of at least 150 mg/g. All CKD patients had ≥6 months of documented medical history to exclude acute kidney injury. Healthy controls met eGFR ≥90 mL/min/1.73 m^2^ with exclusion of chronic conditions (diabetes, hypertension) and body mass index outside 18.5-24 kg/m^2^ range. The study established standardized exclusion criteria to eliminate potential confounding factors, specifically excluding patients with complex renal cysts, renal artery stenosis characterized by a peak systolic velocity (PSV) exceeding 180 cm/s combined with a resistance index (RI) above 0.7, uncontrolled hypertension manifesting as systolic blood pressure greater than 160 mmHg or diastolic pressure surpassing 100 mmHg, and severe cardiac dysfunction classified as NYHA class Ⅲ-Ⅳ. To maintain optimal imaging quality standards, the study further excluded participants demonstrating subcutaneous fat thickness beyond 8 cm or exhibiting ultrasound image stability indices lower than 85%. All enrolled subjects completed thorough clinical evaluations incorporating demographic profiling, comprehensive laboratory testing, and detailed comorbidity analysis, with every case receiving precise staging following the established KDIGO classification system.

### 2.2 Image acquisition

**FIGURE 1:**
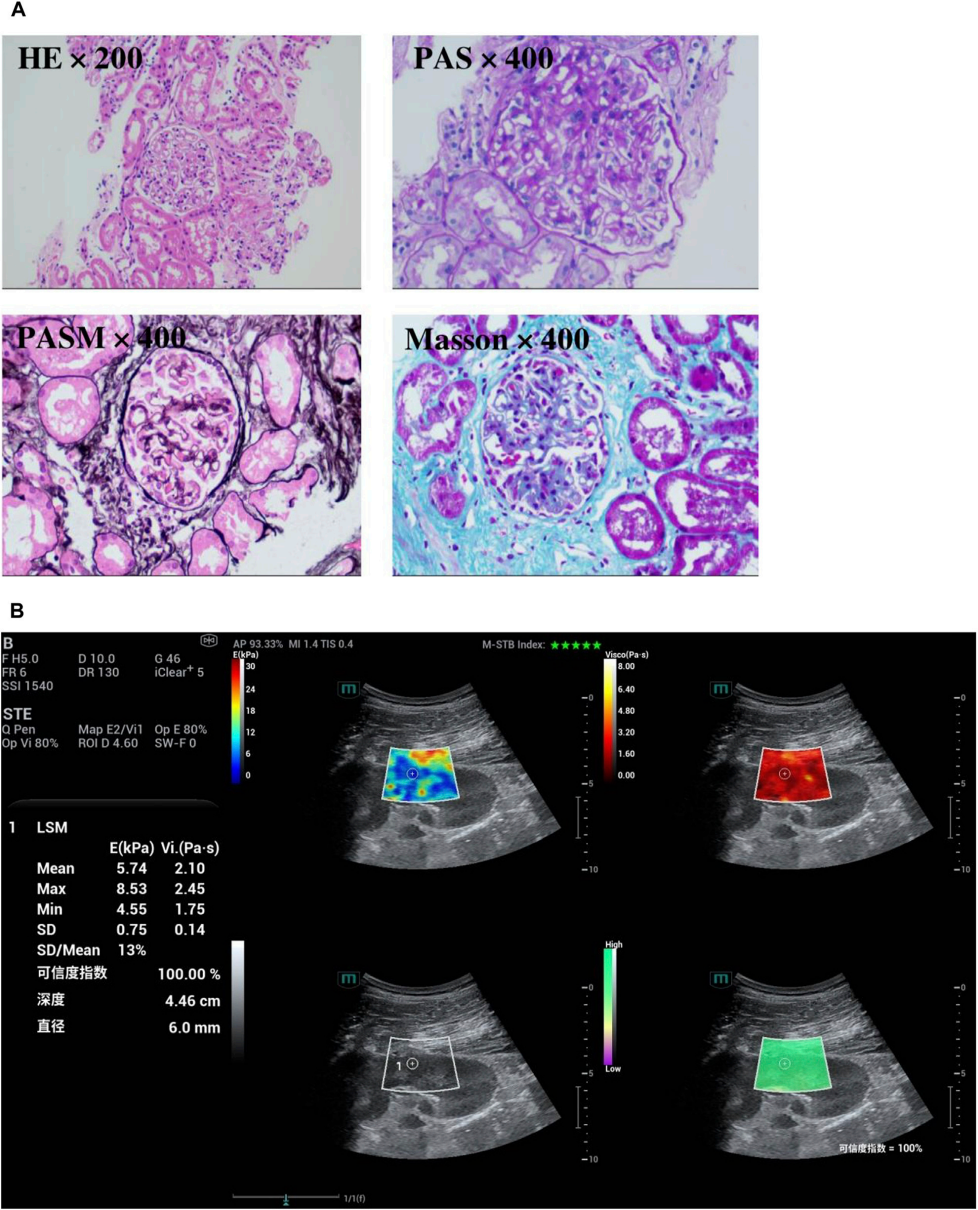
**(A)** Pathology Report: The renal biopsy specimen was routinely processed with HE, PAS, PASM, and Masson trichrome staining. Masson trichrome staining revealed subepithelial fuchsinophilic deposits along glomerular basement membranes, while PASM staining highlighted characteristic spike-like projections on the epithelial side of the GBM. These features are consistent with stage II membranous nephropathy, PLA2R-associated. **(B)** Viscoelastic ultrasound imaging findings in the aforementioned CKD patient with pathological diagnosis: The mean stiffness measured by shear wave elasticity is 5.74 kPa. The mean viscosity coefficient measured by viscoelasticity is 2.10 Pa s.

All renal viscoelastic imaging examinations were performed using the Mindray Resona A20 ultrasound system equipped with an SC7-1U high-frequency transducer (1.2-6.0 MHz) and dedicated viscoelastic analysis module, which simultaneously quantified tissue elasticity and viscosity parameters based on the Voigt biomechanical model, with E mean values reflecting tissue stiffness in kPa and Vi mean values in Pa·s derived from shear wave dispersion characteristics across the 50-400 Hz frequency band. Participants maintained a standardized prone position with arms crossed beneath the forehead for stability during examinations performed by two sonographers with over 5 years of specialized renal ultrasound experience following a double-blind protocol where operators remained unaware of clinical data, beginning with B-mode images of the maximal longitudinal kidney section followed by viscoelastic imaging in the identical plane during which a standardized 2 × 2 cm region of interest was selected within the renal cortex while avoiding vessels and collecting systems during a 6-s breath-hold, with five repeated measurements per kidney recorded for mean values (Figure 1). Stringent quality control criteria including measurement coefficient of variation below 30% within ROI, consistent 6 mm diameter ROI for all acquisitions, and sound wave attenuation correction at 0.5 dB/cm/MHz were implemented to ensure data reliability. Inter-rater reliability was assessed for all ultrasound parameters using intraclass correlation coefficient (ICC) for analysis according to a predefined standardized examination manual.

### 2.3 Statistical analysis

Statistical analyses were performed using R software (version 4.4.3, R Foundation for Statistical Computing, Vienna, Austria). The normality of all continuous variables was rigorously assessed using Shapiro-Wilk tests (n < 50) or Kolmogorov-Smirnov tests (n ≥ 50). Based on these evaluations, continuous variables were expressed as mean ± standard deviation (normally distributed) or median (interquartile range) (non-normally distributed), and compared using independent Student's t-tests or Mann-Whitney U tests. Categorical variables were presented as frequencies (percentages) and analyzed by chi-square or Fisher’s exact tests (for cell counts <5), with statistical significance set at *P* < 0.05.

Variables with >20% missingness were excluded from analysis. When the proportion of missing data exceeds 20%, the remaining data can hardly reflect the true distribution of variables. In particular, non-random missing data will exacerbate bias. In contrast, when the missing proportion is ≤ 20%, the remaining data retain core characteristics. Using 20% as the threshold helps retain valuable variables (detailed missing proportions in Supplementary Table S1) while avoiding interference from low-quality data, thus ensuring statistical power and model stability. For remaining variables, missing data were handled using multiple imputation by chained equations (MICE). Continuous variables were imputed using predictive mean matching (for normally distributed data) or quantile regression (for skewed data), while categorical variables used logistic regression imputation. The study strictly adhered to KDIGO guidelines for clinical data collection from both CKD patients and healthy controls. The analytical framework comprised dependent and independent variables, where the dependent variable represented the CKD diagnosis based on KDIGO criteria (Stevens and Levin, 2013). Independent variables included two categories of measures: conventional ultrasound-measured renal length and viscoelastic parameters, along with basic demographic characteristics (age, sex, and body mass index). All ultrasound examinations were conducted by experienced sonographers with over 5 years of practice following standardized image acquisition and processing protocols to ensure data consistency and comparability throughout the study.

Variable selection was performed using 10-fold cross-validated LASSO regression (repeated 100 times) (Van Beek et al., 2024) to optimize the penalty coefficient λ, where λ.min was selected as the value yielding minimum cross-validated mean squared error (MSE), and λ.1se represented the most parsimonious model within one standard error of the minimum MSE. Variables were retained only if selected in the majority of 1000 bootstrap iterations to ensure stability. Variance inflation factors (VIF) were calculated to confirm the absence of multicollinearity. Model performance was rigorously evaluated through discrimination (AUC with DeLong 95% CI, sensitivity/specificity at Youden-index optimal thresholds), calibration (calibration plots, Brier score and Hosmer-Lemeshow goodness-of-fit test), and clinical utility (decision curve analysis across 0%–100% risk thresholds). Internal validation was enhanced via 1000 bootstrap resamples to derive optimism-adjusted metrics. Results were implemented via a dynamic nomogram explicitly linked to KDIGO staging criteria and therapeutic decision points, with probability thresholds informed by decision curve analysis.

To evaluate the model’s predictive performance across different CKD progression stages, we implemented a multiclass ROC analysis framework with the following specifications: (1) Stage classification followed KDIGO guidelines (Class 1: normal controls; Class 2: G1-3 stages; Class 3: G4-5 stages); (2) Multiclass handling employed one-vs-rest (OvR) methodology to generate stage-specific ROC curves, reporting both sensitivity (True Positive Rate) and 1-specificity (False Positive Rate) metrics; (3) Comprehensive evaluation included micro-averaged AUC for overall discriminative ability alongside stage-specific AUC values. All performance metrics were derived with 1000 bootstrap iterations to generate 95% confidence intervals, ensuring robust variance estimation.

## 3 Results

### 3.1 Baseline characteristics of study participants

Following data preprocessing to handle missing values, the final analysis included 228 participants comprising 138 chronic kidney disease (CKD) patients and 90 healthy controls. Regarding the imbalance in sample size between the case group (138 cases) and the control group (90 cases), we implemented targeted statistical adjustments: robust standard errors were used in the logistic regression analysis to reduce the impact of inter-group sample size differences on the variance of parameter estimates. Furthermore, the model calibration curve showed that the mean absolute error between predicted probabilities and actual observed probabilities was 0.03 (<0.05), which further confirms that the calibration effect of the model under this sample structure is acceptable. All intraclass correlation coefficients (ICC) for measurements exceeded 0.8, indicating excellent intra-observer reliability (Supplementary Table S2). As shown in Table 1, comparative analysis of renal ultrasound parameters revealed significant intergroup differences across multiple indicators. CKD patients exhibited markedly reduced renal dimensions bilaterally compared to controls (left kidney: 9.76 ± 1.42 cm vs. 10.32 ± 0.88 cm; right kidney: 9.73 ± 1.42 cm vs. 10.23 ± 0.68 cm; both *P* < 0.001), consistent with the characteristic renal atrophy observed in CKD progression and confirming the diagnostic value of renal length measurements. Regarding tissue characterization parameters, the CKD group demonstrated significant histopathological alterations with elevated elastic moduli bilaterally (left kidney E mean: 6.02 ± 1.33 kPa vs. 5.34 ± 0.50 kPa; right kidney: 6.07 ± 1.17 kPa vs. 5.36 ± 0.34 kPa; both *P* < 0.001) and increased shear wave velocities (left kidney Cs mean: 1.39 ± 0.14 m/s vs. 1.32 ± 0.06 m/s; right kidney: 1.40 ± 0.12 m/s vs. 1.33 ± 0.04 m/s; both *P* < 0.001), suggesting increased renal tissue stiffness potentially associated with fibrotic processes. Most notably, viscosity parameters (Vi mean) reflecting inflammatory activity showed significant elevation in CKD patients (1.99 ± 0.31 Pa·s vs. 1.64 ± 0.11 Pa·s bilaterally, *P* < 0.001), providing quantitative biomarkers for renal inflammation assessment.

**TABLE 1:** Baseline characteristics of patients with CKD group and control group.

Characteristics		CKD group (n = 138)	Control group (n = 90)	*P*
Left Kidney length (cm)		9.76 ± 1.42	10.32 ± 0.88	<0.001
Right Kidney length (cm)		9.73 ± 1.42	10.23 ± 0.68	<0.001
Left Kidney E Mean (KPa)		6.02 ± 1.33	5.34 ± 0.50	<0.001
Right Kidney E Mean (KPa)		6.07 ± 1.17	5.36 ± 0.34	<0.001
Left Kidney Cs Mean (m/s)		1.39 ± 0.14	1.32 ± 0.06	<0.001
Right Kidney Cs Mean (m/s)		1.40 ± 0.12	1.33 ± 0.04	<0.001
Left Kidney Vi Mean (Pa·s)		1.99 ± 0.31	1.64 ± 0.11	<0.001
Right Kidney Vi Mean (Pa·s)		1.99 ± 0.32	1.65 ± 0.12	<0.001
Age (years)		49.77 ± 16.92	41.36 ± 10.76	<0.001
BMI (Kg/m^2^)		24.05 ± 3.55	24.34 ± 3.79	0.457
Gender	Female	58 (42.0%)	46 (51.1%)	0.178
	Male	80 (58.0%)	44 (48.9%)	

Data are presented as means ± standard deviations for continuous variables and numbers (percentages) for categorical variables. BMI, is Body Mass Index. The Student’s t-test was used for continuous variables, and the chi-square test was used for categorical variables.

Demographic analysis revealed CKD patients were older (49.77 ± 16.92 years vs. 41.36 ± 10.76 years, *P* < 0.001) with comparable BMI between groups (24.05 ± 3.55 kg/m^2^ vs. 24.34 ± 3.79 kg/m^2^, *P* = 0.457), effectively excluding body mass index as a confounding factor. Gender distribution analysis showed female proportions of 42.0% (58/138) in CKD and 51.1% (46/90) in control groups (*P* = 0.178), with male predominance (58.0%) observed in CKD patients. Despite these non-significant demographic differences, both BMI and gender were incorporated as covariates in subsequent modeling to ensure the identified ultrasound parameter differences specifically reflected CKD-related pathological changes rather than demographic variations.

### 3.2 Variable selection and model construction

**FIGURE 2:**
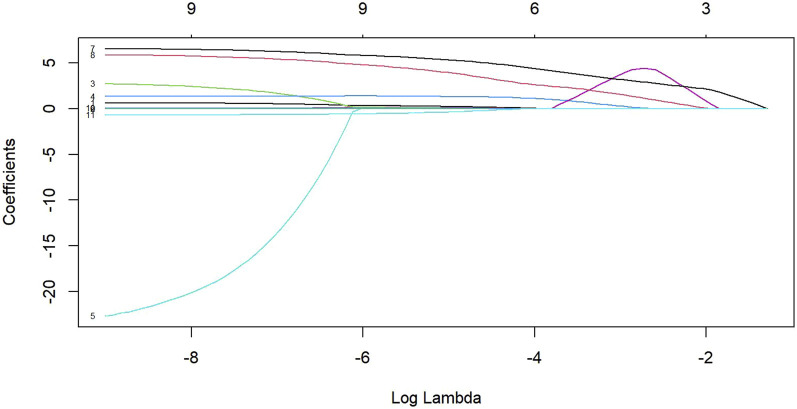
LASSO Regression Coefficient Path Diagram. The plot displays how feature coefficients shrink as regularization intensity increases (left to right). Numbers along the right margin (3, 6, 9,…) indicate the count of non-zero coefficients at selected λ values.

**FIGURE 3:**
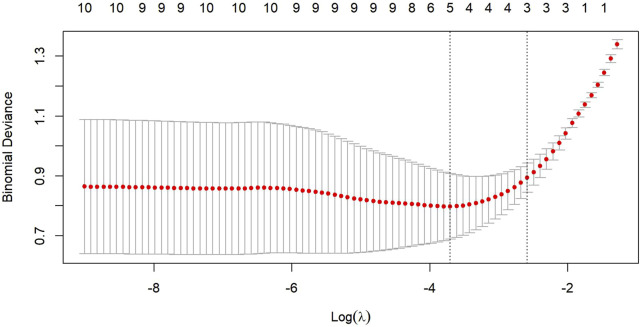
Regularization path plot for binomial deviance across different penalty terms (log(λ)). The x-axis represents the natural logarithm of regularization strength (λ), while the y-axis shows the binomial deviance. The numbers along the curve (10, 10, 9,…) indicate the number of non-zero coefficients retained at each λ value.

The LASSO regression analysis (Figures 2, 3) demonstrated that as the penalty parameter λ increased, the coefficients of each parameter gradually shrank toward zero. The model achieved optimal performance at log(λ) = −3.705, retaining four significant variables. As λ decreased, the number of variables systematically reduced from 10 to 0, confirming the stability of the variable selection process. Among the renal ultrasound parameters, the viscosity coefficients of both kidneys, the elasticity parameter of the right kidney (RE) and age were retained in the final model. Multicollinearity assessment confirmed all retained variables had Variance Inflation Factors (VIF) < 5 (Supplementary Table S3). Notably, the viscosity parameters (LVi/RVi) exhibited the highest absolute coefficient values (Table 2), indicating their dominant contribution to CKD diagnosis. In contrast, age showed the smallest absolute coefficient, suggesting its limited independent diagnostic value after adjusting for other factors.

**TABLE 2:** Regression model variable coefficients tabl**e**.

Index	Coefficient
Intercept	−25.627801
Right Kidney E Mean (KPa)	1.536479
Left Kidney Vi Mean (Pa·s)	5.980906
Right Kidney Vi Mean (Pa·s)	3.789646
Age (years)	0.009335

The 5 selected features and their coefficients.

### 3.3 Model diagnostic performance

**FIGURE 4:**
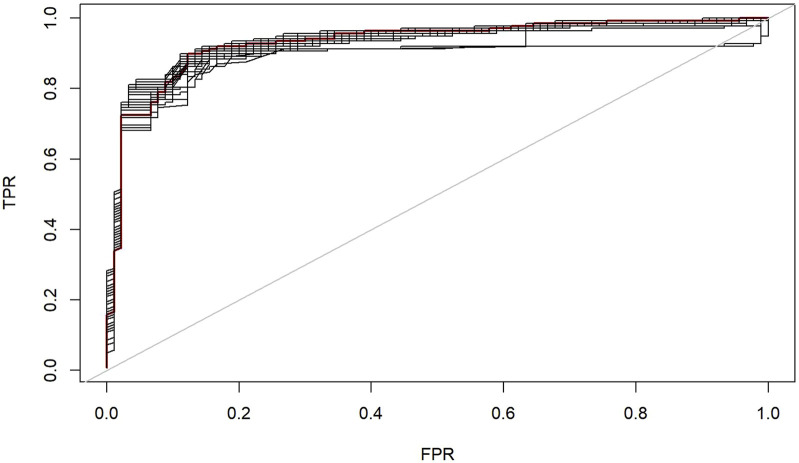
Sensitivity-specificity Balance Curve. Receiver operating characteristic (ROC) curve analysis showing the trade-off between TPR and FPR at different classification thresholds. The TPR values (sensitivity) are highlighted at 0.2, 0.4, 0.6, and 0.8 intervals, demonstrating the model’s performance across various decision boundaries.

**FIGURE 5:**
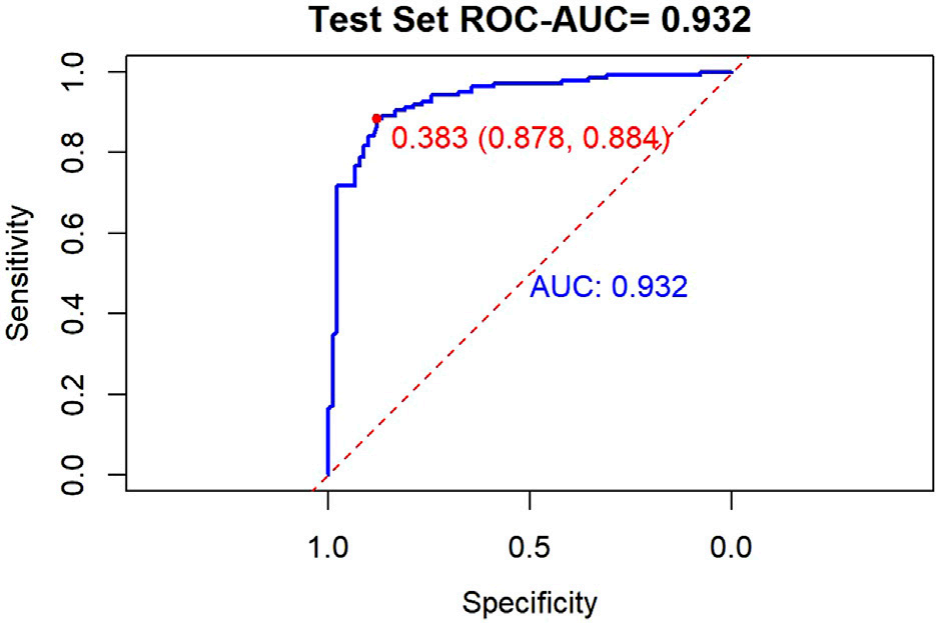
ROC Curve of the Test Set. The optimal diagnostic cut-off value of the prediction model was 0.383, with an area under the curve (AUC) of 0.932.

ROC analysis of the training set revealed excellent diagnostic performance (Figure 5), with an AUC of 0.932 (95% CI: 0.908–0.956). At the optimal threshold determined by Youden’s index, the sensitivity was 87.8% (80.0%-93.3%) and specificity was 88.4% (80.2%-94.0%). The ROC curve (Figure 4) clearly illustrated the relationship between the true positive rate (TPR) and false positive rate (FPR) across different thresholds, demonstrating the model’s ability to maintain high TPR while effectively controlling FPR. In the validation set (Figure 5), at a cutoff of 0.383, sensitivity and specificity were both 0.884 and 0.878, respectively. The TPR-FPR curve further indicated that the model achieved TPR >0.8 at FPR <0.2, outperforming the random guessing line.

### 3.4 Model calibration and clinical utility analysis

**FIGURE 6:**
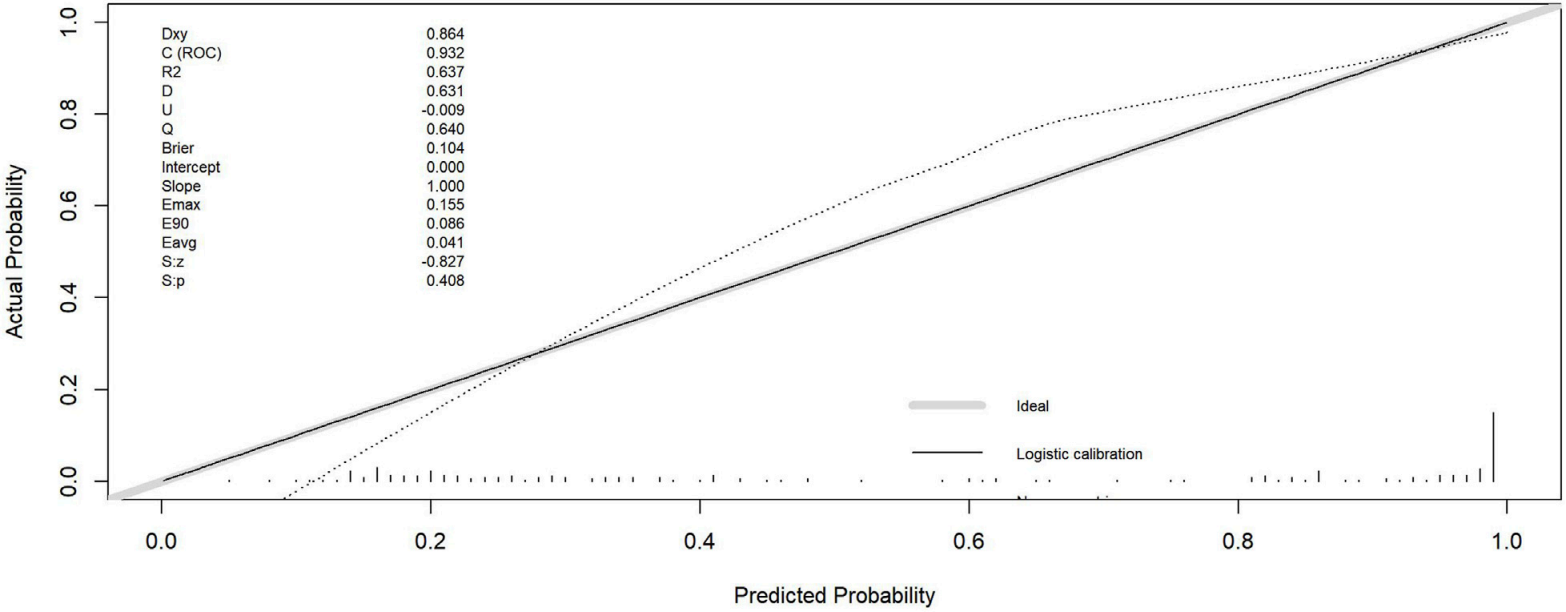
Calibration Curve and Metrics. The y-axis represents the actual probability of CKD, and the x-axis represents the predicted probability. Our model demonstrates good consistency between predicted and actual probabilities.

**FIGURE 7:**
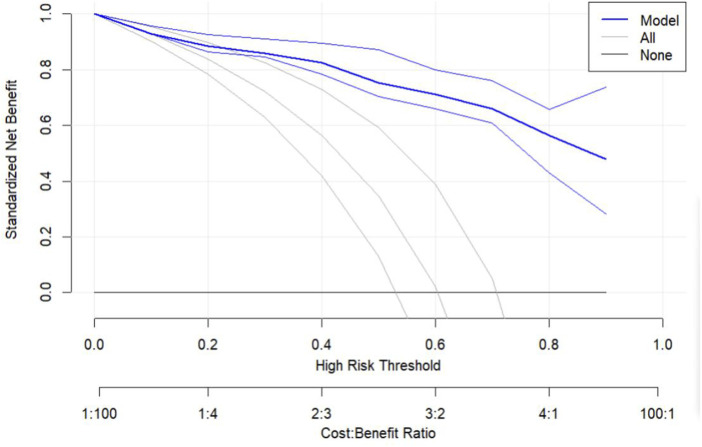
Decision Curve Analysis of the Predictive Model. The y-axis represents the standardized net benefit, and the x-axis indicates the high-risk threshold probability. The solid line denotes the model’s net benefit, while the gray dashed lines represent the “treat all” and “treat none” strategies. The model demonstrates clinical utility between 20% and 80% risk thresholds, with peak net benefit at 40%.

Calibration analysis (Figure 6; Table 3) demonstrated strong agreement between predicted probabilities and observed outcomes. The diagnostic model exhibited outstanding discriminative ability, with an AUC of 0.932, and explained approximately 63.7% of the outcome variance (R^2^ = 0.637), confirming its high diagnostic accuracy. Calibration metrics indicated close alignment between logistic calibration curves, nonparametric calibration curves, and the ideal calibration line in the intermediate-risk range (actual probability = 0.4). (Supplementary analysis: Hosmer-Lemeshow goodness-of-fit test indicated potential departure from perfect calibration [P = 0.001], though this may be influenced by the test’s sensitivity to group partitioning and large sample size, consistent with other calibration evidence.) The Brier score of 0.104 suggested low overall prediction error, while the calibration intercept of 0.000 indicated slight systematic overestimation. A calibration slope of 1.000 suggested potential mild overfitting; however, the model maintained good accuracy across most risk ranges, with minor overestimation in the low-risk range (predicted probability: 0–0.3) and slight underestimation in the high-risk range (E90 = 0.086). Decision curve analysis (Figure 7) further confirmed clinical utility, demonstrating superior net benefit versus “treat-all” and “treat-none” strategies at risk thresholds of 20%-80%, with peak utility at 40% threshold probability. These results collectively confirm that the ultrasound-based diagnostic model provides well-calibrated risk predictions while maintaining excellent discriminative performance.

**TABLE 3:** Summary of diagnostic performance metrics for the CKD prediction mode**l**.

Metric	Value	Interpretation
Dxy	0.864	Strong predictive separation
AUC (C-statistic)	0.932	Excellent diagnostic accuracy
Brier score	0.104	Good overall prediction accuracy
Calibration intercept	0.000	Minor systematic overestimation
Calibration slope	1.000	Slight overfitting
E90	0.086	Error in high-risk estimates

AUC is area under the receiver operating characteristic curve; E90 is 90th percentile absolute error.

### 3.5 Risk scoring system

**FIGURE 8:**
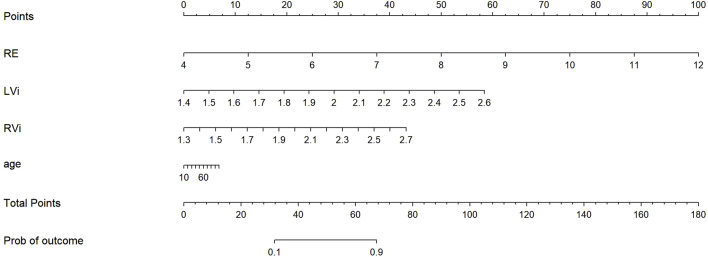
Schematic Diagram of the Risk Scoring System. Based on sonographic examination, a nomogram was established in the primary cohort incorporating the following variables: right kidney E mean (RE), left kidney Vi mean (LVi), right kidney Vi mean (RVi) and age. Each variable was assigned corresponding predictor points according to the point scale marked at the top. The points of all variables were summed, and the total points were then projected onto the bottom scale to determine the predicted probability of CKD.

A visualized CKD risk scoring system was developed based on the selected ultrasound parameters (Figure 8). This integrated scoring system incorporates multidimensional diagnostic indicators, where the total score is calculated as: Total Points = RE (renal elasticity) + LVi/RVi (viscosity parameters) + age. Each parameter was assigned a specific weight according to its regression coefficient, and the total score was converted into a CKD probability ranging from 0.1 to 0.9 via a linear predictor using the logistic function specified in Supplementary Material Equation. The scoring system highlights the significant contribution of viscosity parameters, which reflect renal inflammatory activity, along with elasticity (RE) and age, forming a comprehensive multidimensional evaluation framework. Notably, the viscosity parameters alone could contribute over 30 points to the total score, reaffirming their pivotal diagnostic value in CKD assessment.

### 3.6 Model discriminative performance

**FIGURE 9:**
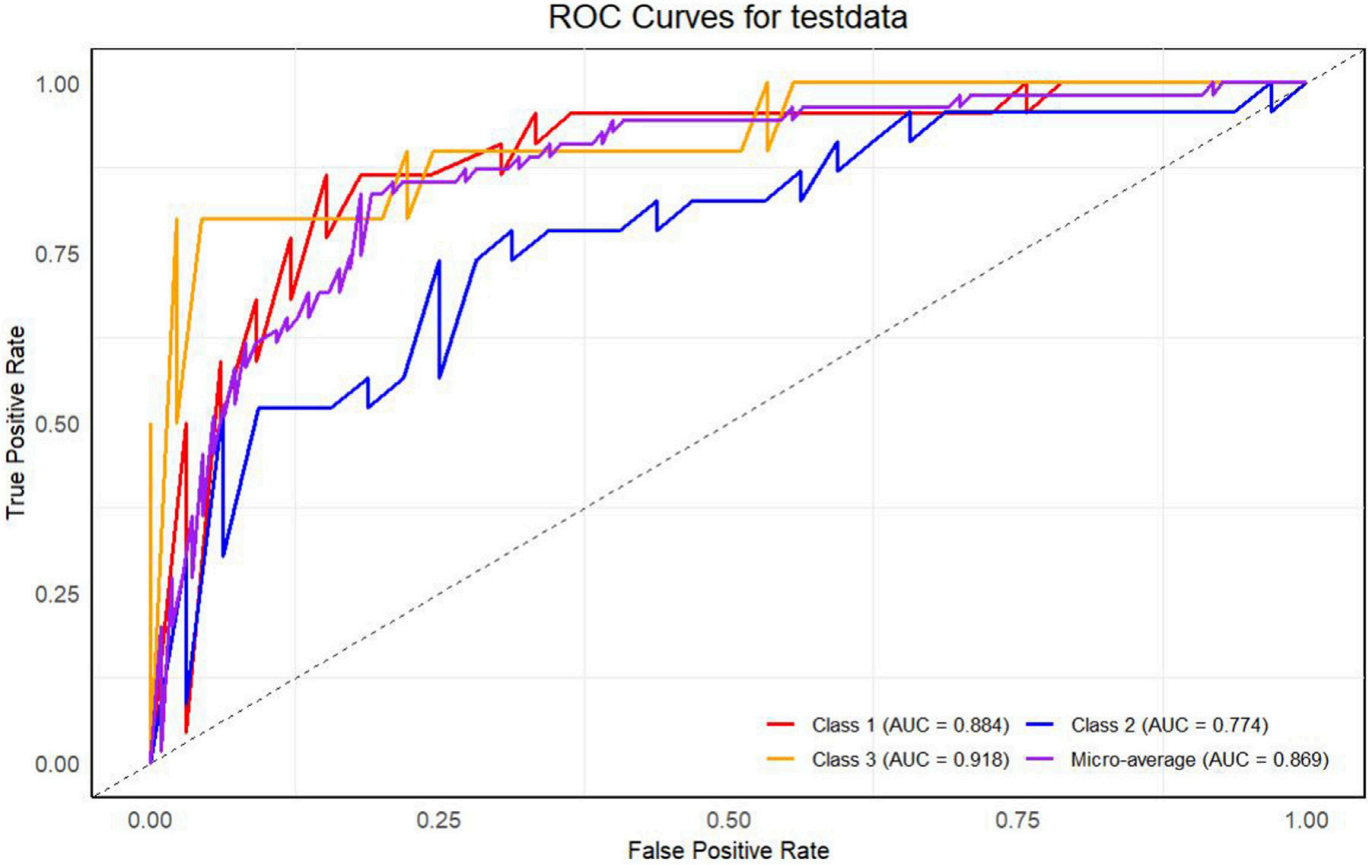
Multiclass ROC Analysis of CKD Staging Performance. Stage-specific ROC curves demonstrating diagnostic accuracy for: Class 1 (normal controls, AUC = 0.884), Class 2 (G1-3 CKD, AUC = 0.774), and Class 3 (G4-5 CKD, AUC = 0.918). Micro-average ROC curve (AUC = 0.869) representing overall classification performance.

ROC analysis of the test set (Figure 9) revealed excellent stage-specific diagnostic accuracy. The model achieved highest discrimination for end-stage CKD (Class 3: AUC = 0.918, 95% CI: 0.890–0.946), followed by normal controls (Class 1: AUC = 0.884, 95% CI: 0.850–0.918) and early-stage CKD (Class 2: AUC = 0.774, 95% CI: 0.730–0.818). DeLong’s test confirmed significant AUC differences between Class 3 vs Class 2 (*P* < 0.001). The micro-average AUC (0.869) indicated consistent overall performance, supporting clinical utility in distinguishing high-risk patients.

## 4 Discussion

This study systematically analyzed renal ultrasound parameter differences between CKD patients and healthy controls to develop a diagnostic model based on multimodal ultrasound indicators, providing a novel approach for noninvasive CKD diagnosis. The results not only validated the diagnostic value of multiple ultrasound parameters for CKD but also elucidated their specific roles in reflecting distinct renal pathological changes, demonstrating significant clinical implications.

This study confirmed the continued diagnostic value of traditional renal length measurements in CKD. We observed significantly shorter kidney lengths in CKD patients (*P* < 0.001), reflecting the pathological processes of nephron loss and renal atrophy during disease progression (Singla et al., 2022). However, relying solely on kidney length measurements has limitations, particularly in early-stage disease (Leong et al., 2018). To address this, we innovatively incorporated novel ultrasound parameters into the diagnostic framework, among which the viscosity parameter (Vi) demonstrated the highest diagnostic contribution, a finding consistent with the positive GFR-viscosity relationship reported by Maralescu et al. (2022) and further validating prior research on inflammation-induced increases in tissue viscoelasticity. Additionally, as per the study by Yuan et al. (2024), new tissue viscosity indices show advantages over traditional elastic modulus in differentiating CKD from normal renal function. The elastic modulus showed significant elevation (12.7% in the left kidney and 13.2% in the right kidney, both *P* < 0.001), while the shear wave velocity (Cs) increased by approximately 5.3%, objectively reflecting the degree of renal fibrosis, aligning with Wang et al.’s (Arndt et al., 2010; Lukenda et al., 2014; Nakao et al., 2015) histologically confirmed observations of increased tissue stiffness in CKD. These novel parameters provide quantitative biomarkers for assessing pathophysiological changes in CKD, effectively addressing the limitations of conventional ultrasonography.

The CKD diagnostic model developed in this study offers significant technical and clinical advantages. Through LASSO regression, key parameters were selected, including renal elasticity (RE), viscosity (LVi/RVi), and age. These parameters demonstrate exceptional discriminative ability, with an AUC of 0.932, while effectively avoiding overfitting (calibration slope: 1.000). This represents a notable improvement over previous single-parameter diagnostic methods. For instance, in studies evaluating renal fibrosis using shear wave elastography, the reported AUC reached only 76.4% (Chen et al., 2024). The enhanced performance primarily stems from the synergistic integration of multiparametric data. Viscosity parameters, which carry the highest weight and contribute approximately 81.5%, are sensitive to early inflammatory changes. In contrast, elasticity (RE) specifically reflects the severity of fibrosis (Chen et al., 2024; Barr, 2006; Hossain et al., 2018). Clinically, the innovative visual nomogram (Figure 8) converts complex ultrasound measurements into an intuitive 0–160-point scale, which corresponds to CKD probabilities ranging from 0.1 to 0.9. Notably, the parameter weights are scientifically aligned with their pathological relevance, underscoring the model’s clinical utility and biological plausibility. The optimal cutoff of 0.383 balances high sensitivity (0.884) and specificity (0.878), making it suitable for both population screening to identify high-risk individuals and specialist diagnosis to confirm CKD in ambiguous cases. Context-specific adjustments to decision thresholds are recommended: a lower threshold may enhance early detection sensitivity in high-risk populations such as patients with diabetes or hypertension, while a higher threshold can reduce overdiagnosis and unnecessary follow-up in low-risk settings like routine check-ups. Decision curve analysis (DCA) showed that the 40% high-risk threshold is the optimal decision threshold. However, its clinical application needs to be adjusted individually according to patient staging and scenarios: in early-stage CKD, the threshold should be raised to 60%-80% to avoid over-intervention; in advanced-stage CKD, the threshold can be lowered to 20%-40% to prioritize the initiation of active management. To realize the clinical translation of the 40% threshold, it is recommended that individuals with a risk of ≥40% should be included in the high-frequency monitoring pathway; those with ≥40% risk complicated with diabetes/hypertension should be rapidly referred to nephrology specialists; and patients with ≥40% risk accompanied by proteinuria should be initiated on evidence-based treatments such as SGLT2 inhibitors. To ensure accurate implementation, clinician training should focus on translating the 0–160-point scale to probability estimates and applying context-adjusted thresholds through case-based simulations to standardize interpretation and minimize inter-operator variability. The ROC analysis revealed clinically meaningful stage-dependent performance, with exceptional discrimination for end-stage CKD (G4-5 AUC = 0.918) reflecting distinct sonographic signatures of advanced fibrosis, while the moderate early-stage detection (G1-3 AUC = 0.774) aligns with known challenges in identifying initial microstructural changes. The robust overall performance (micro-average AUC = 0.869) surpasses clinical adoption thresholds and demonstrates advantages over conventional biomarkers, while maintaining the accessibility benefits of ultrasound compared to advanced imaging modalities. In advanced CKD (G4-G5), its high discriminative power (AUC 0.918) supports utility as a decision aid replacing invasive biopsies; whereas in early-stage CKD (G1-G3), it should be positioned as a complementary tool to eGFR—identifying subclinical high-risk patients (e.g., those with eGFR>60 mL/min/1.73 m^2^ but model-predicted risk ≥40%) to initiate early renoprotective therapy. Recent breakthroughs in urinary peptidomics technology (test set AUC 99%) (Li et al., 2023) demonstrate the potential of molecular diagnostics for CKD detection. Our study confirms that ultrasound viscoelastic parameters achieve comparable discriminative power in advanced CKD (AUC 0.918), with unique clinical value characterized by real-time, cost-effective, and highly reproducible advantages. The two technologies are inherently complementary: peptidomics excels in capturing early molecular abnormalities, while ultrasonography quantifies late-stage structural remodeling.

Compared to traditional GFR-dependent diagnostics, this model offers a fully noninvasive approach that overcomes GFR’s hemodynamic variability and remains unaffected by short-term factors like diet or hydration status, enabling more stable organ-level assessments critical for long-term monitoring of disease progression and treatment response. The STVi model complements the KDIGO 2024 guidelines by providing structural kidney assessment in cases where traditional biomarkers (eGFR/ACR) are inconclusive (e.g., early CKD with normal eGFR but abnormal STVi). Clinically, STVi may serve as an adjunct tool when ACR is unavailable (e.g., resource-limited settings) or unreliable (e.g., non-proteinuric CKD). Future studies should validate STVi in multicenter cohorts integrating ACR and established risk factors, assess cost-effectiveness, and explore automated analysis to reduce operator dependence. STVi’s real-time imaging may benefit high-risk monitoring (e.g., diabetes) and settings requiring rapid repeat assessments. Furthermore, the multiparametric ultrasound assessment not only matches GFR’s diagnostic efficacy (AUC 0.932) but also provides complementary structural information unattainable through GFR alone. Their integration pioneers a “function-structure” integrated diagnostic paradigm for precision nephrology.

However, this study has several limitations. First, as a single-center investigation, the sample representativeness may be limited. Second, although the viscosity parameter demonstrated excellent diagnostic performance, its measurement standardization requires further refinement. Additionally, the model’s performance variations across different CKD stages need to be validated in subsequent studies. Future research should focus on conducting multicenter validation studies to confirm the model’s generalizability across diverse populations and settings, optimizing ultrasound parameter measurement protocols to enhance reproducibility for consistent data collection, and exploring the model’s potential applications in predicting CKD progression and evaluating therapeutic efficacy to inform clinical decision-making and treatment strategies.

## 5 Conclusion

The sound touch viscosity has emerged as a valuable noninvasive diagnostic tool that effectively evaluates renal fibrosis, overcoming the limitations of traditional kidney biopsies such as invasiveness and complication risks. This advancement optimizes clinical management for CKD patients by facilitating early identification of high-risk cases and enabling timely interventions. The developed nomogram incorporating viscoelastic parameters significantly improves diagnostic accuracy for renal pathology in CKD patients while demonstrating potential for predicting kidney-related clinical outcomes, particularly in resource-limited settings. With its noninvasive nature and high diagnostic performance, this technique represents a clinically valuable tool for both diagnosis and prognosis assessment in routine practice.

## Data Availability

The original contributions presented in the study are included in the article/Supplementary Material, further inquiries can be directed to the corresponding author.
