# Supplementary material for: A clinically applicable CKD diagnostic model derived from sound touch viscosity ultrasound and LASSO regression
Source: Front Bioeng Biotechnol. 2025 Sep 22;13:1651500. doi: 10.3389/fbioe.2025.1651500 (PMC12498016; doi:10.3389/fbioe.2025.1651500)
Supplement: Supplementary file 1 [file Table1.docx]

**Supplementary appendix**

**Supplemental text**

***Data processing***

**Supplementary Table 1. Missing Data Proportion of Key Variables**

| **Variables** | Total Sample Size | Number of Missing Cases | Missing Proportion (%) |
| --- | --- | --- | --- |
| Left Kidney length (cm) | 247 | 18 | 7.9 |
| Right Kidney length (cm) | 239 | 7 | 3.1 |
| Left Kidney E Mean (KPa) | 246 | 13 | 5.7 |
| Right Kidney E Mean (KPa) | 247 | 19 | 8.3 |
| Left Kidney Cs Mean (m/s) | 248 | 13 | 5.7 |
| Right Kidney Cs Mean (m/s) | 251 | 19 | 8.3 |
| Left Kidney Vi Mean (Pa·s) | 246 | 16 | 7.0 |
| Right Kidney Vi Mean (Pa·s) | 228 | 19 | 8.3 |
| Age (years) | 237 | 6 | 2.6 |
| BMI (Kg/m^2^) | 257 | 27 | 11.8 |
| Gender | 233 | 3 | 1.3 |

**Table 2. ICC Reliability of Renal Ultrasound Parameters**

| **Variables** | ICC | 95% CI of ICC |
| --- | --- | --- |
| Left Kidney length (cm) | 0.899 | 0.814 < ICC < 0.939 |
| Right Kidney length (cm) | 0.871 | 0.835 < ICC < 0.899 |
| Left Kidney E Mean (KPa) | 0.863 | 0.807 < ICC < 0.901 |
| Right Kidney E Mean (KPa) | 0.879 | 0.683 < ICC < 0.939 |
| Left Kidney Cs Mean (m/s) | 0.821 | 0.774 < ICC < 0.860 |
| Right Kidney Cs Mean (m/s) | 0.811 | 0.759 < ICC < 0.853 |
| Left Kidney Vi Mean (Pa·s) | 0.864 | 0.807 < ICC < 0.903 |
| Right Kidney Vi Mean (Pa·s) | 0.909 | 0.884 < ICC < 0.929 |

**Supplementary** **Table 3. VIF Values of Each Variable**

| Index | VIF |
| --- | --- |
| Right Kidney E Mean (KPa) | 1.108765 |
| Left Kidney Vi Mean (Pa·s) | 1.967171 |
| Right Kidney Vi Mean (Pa·s) | 2.188991 |
| Age (years) | 1.128463 |

***Supplementary analysis: Details of the Hosmer-Lemeshow Goodness-of-Fit Test***

Hosmer-Lemeshow goodness-of-fit test indicated potential departure from perfect calibration [P = 0.001], though this may be influenced by the test's sensitivity to group partitioning and large sample size, consistent with other calibration evidence.

In terms of consistency, there is a certain discrepancy between this result and the main calibration indicators (calibration curve, Brier score, calibration intercept, and slope). The calibration curve showed that the model highly overlaps with the ideal line in the intermediate-risk range (predicted probability around 0.4). The Brier score (0.104) is at a low level, suggesting a small overall prediction error. Moreover, the calibration intercept (0.000) and slope (1.000) further support that the model has no obvious systematic bias. This discrepancy may stem from the sensitivity of the model.

In conclusion, the single result of the Hosmer-Lemeshow test needs to be interpreted comprehensively in conjunction with other calibration indicators. The core calibration performance of the model in this study is still based on the visual calibration curve and quantitative error indicators (Brier score). The Hosmer-Lemeshow test result is only used as an auxiliary reference, indicating that the model may have slight biases in the extreme risk ranges (e.g., predicted probability < 0.1 or > 0.9), but it does not affect the reliability of the overall calibration effect.​

***Supplementary Material Equation:***

Logistic Regression Model Formula

Mathematical expression:

$logit(CKD)=\beta_{0}+\beta_{1}X_{1}+\beta_{2}X_{2}+$⋯+$\beta_{n}X_{n}$

$\beta_{0}$ is the intercept term, representing the log-odds when all independent variables are zero.

$\beta_{i}$ is the regression coefficient for the i-th predictor, which indicates the change in log-odds of CKD per unit increase in that variable.

$X_{i}$ is the value of the i-th independent variable (e.g., age, estimated glomerular filtration rate, etc.).

Probabilities are derived from log-odds through the logistic function (sigmoid function):

$$P(CKD=1)=\frac{1}{1+e^{-logit(CKD)}}$$
